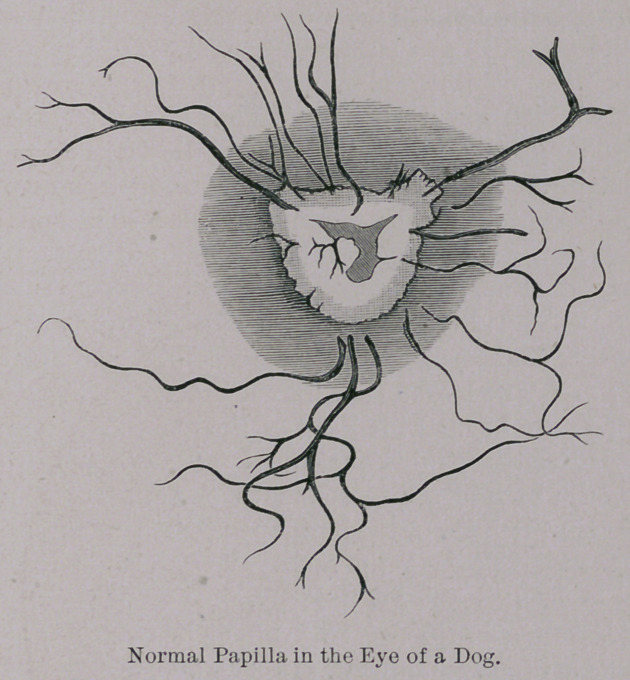# Neuroretinitis Optica

**Published:** 1884-04

**Authors:** 


### Art. XIII.—NEURORETINITIS OPTICA.

(The following article is not intended to be a compilation in any sense of the word, but is a translation of selected portions of the writings of several authors.)

SCHWEIGGER*, says: "With the word Neuroretinitis or Retinitis, we distinguish those cases in which the opthalmoscopic changes are concentrated in and around the bulb, or entrance of the optic nerve in the fundus of the eye. The conditions revealed by opthalmoscopic examination in such cases are Hyperaemia, cloudiness and tumefaction of the diseased tissues."

"Hyperaemia is most marked in the veins of the retina in immediate apposition with the optic bulb; these vessels appear distended and to have assumed a markedly serpentine course."

The optic bulb assumes a decidedly red appearance in consequence of the increased development of the numerous minute vessels in this portion of the nerve; this red shade assumes a peculiar lilac color at times which extends to the appositional portions of the retina.

"The clouded condition of the tissues of the nerve and the adjacent parts of the retina causes the more profoundly seated parts, the lamina cribosa, and the outlines of the nerve and vessels of the retina, to appear as if covered by a delicate veil, or they may be entirely obscured from sight."

"The tumefaction of the optic nerve may be proportionally less than that of the retina, so that the latter appears to enclose the bulb as if walled round, but as a rule the swollen condition is evenly distributed over all the complicated tissues, frequently to such a degree that the bulb perceptibly projects beyond the level of its surroundings."

*Augenheilkunde.

The extent of the swollen condition of the optic nerve is to be determined in the same manner, by means of the opthalmoscope, as is adopted in the study of like conditions in other parts of the fundus. This condition can be most easily determined by observing the deportment of those retinal vessels which ascend over the contours of the bulb and are lost in its tissues: these vessels being pressed prominently outward—forward—are easily to be seen in the perpendicular picture, while in the transverse picture they appear to suffer a marked paralletic displacement in comparison with the retinal vessels in their immediate vicinity. The serpentine course of the vessels in the retina frequently extends to some distance into the body of that membrane, and when plentiful, leads one to assume that oedema is present.

" Haemorrhagic points in the vicinity of the optic nerve are occasionally to be seen. White spots appear in the retina, or in the bulb itself, as a consequence of circumscribed sclerotic degeneration of the nerve fibres, or of fatty degeneration of the same: peculiar stellate points are sometimes to be seen in the macula lutea."

"As the disease progresses, the swollen condition of the optic bulb recedes; the redness diminishes in intensity, though the clouded appearance still remains, giving to the bulb a greyish red or whitish appearance: these changes also extend, in a less degree, to the limiting parts of the retina. In some cases I have, at this stage, seen a peculiar serpentine condition appear in the most delicate retinal veins on the surface of and in the vicinity of the nerve. Sometimes slight changes in the choroid may be seen in the vicinity of the optic nerve after the tumefaction of the bulb has receded and the cloudiness disappeared from the retina, which is to be sought in the increased dimensions of the intraocular portion of the nerve, and the proliferation occurring in the internal (outer) layers of the retina.

" The sight is decidedly diminished in most cases, and as a rule important defects in the field of vision also appear at the same time. We frequently see considerable disturbances in the sight appear in the course of the disease without being able to discover any corresponding pathological disturbances with the opthalmoscope; or severe changes may be apparent with only slight visual alterations. A remarkable phenomenon is, that by retention of the sight, transitory darkening of the field of vision suddenly occurs."

"Sometimes the visual disturbances develop gradually while at others they appear with astonishing rapidity, so that blindness results in a very few hours."

"Neuritis can appear as an idiopathic disturbance without any other complication being visible. Occasionally, contusion of the orbital region may give rise to the disturbance. Constitutional disturbances sometimes act as a cause of the disease,such as changes in the circulation in distant organs,anomalies in the menstruation, syphilis and lead poisoning. In other cases we may be able to demonstrate disturbances in the orbital or intracranial course of the nerve, such as tumors or inflammatory processes, other intracranial diseases as meningitis, encephalomalatic centres, tumors in the brain are connected with neuroretinitis. These conditions cannot be safely conjectured by opthalmoscopic examination, to be at the bottom of the disturbances.

### Neuroretinitis in the Dog.*

The first case that came to my observation was in a poodle. The owner could give no authentic data with reference to the origin of the disease in the eye, although he had noticed that the sight had been poor for years : for four or five years he thought it had become worse.

Status presens : The patient is totally blind ; the lids appear normal ; the palpebral portion of the conjunctiva is slightly injected. The cornea is perfectly transparent in all its parts, as well as the contents of the anterior chamber. The pupil is evenly distended in its circumferences, but does not react. A greyish-clouded appearance of the lens can be seen in the vicinity of the posterior pole, as well as in its superior interior portion. The vitreous humor is normal.

Before giving the results of the opthalmoscopic examinations it seems justifiable to make a few remarks upon the normal picture which that instrument gives of this portion of the fundus of the eye in the dog.

The form of the optic disc—papilla—is not exactly as described by Bayer—roundish or oval, but more nearly resem-

*Dr. R. Westrum, Zeitshrift für Vergleichende Augenheilkunde, Vol. 1.

bles a right angled triangle, the angles at the base being somewhat rounded, corresponding to the inferior edges of the entrance of the optic nerve. The superior point of union of the two sides is not too sharply outlined, the entrance of the optic nerve at this point separating in several fine bundles. The veins and arteries are to be distinguished as well by their difference in color as in their dimensions. Those vessels which take a superior course appear singly, while those that take a lateral and inferior course frequently have a common stem from which they bifurcate. A slight indentation may be seen in the middle of the papilla which is caused by the radiating course which the nerve fibres take from this point, also called physiological excavation ; this, however, bears no comparison with the deeper excavation frequently met with in man. The disc in the dog, therefore, has the appearance of very gradually inclining from its peripheral to its central parts. An irregular oval appearance of the disc is, however, occasionally met with. The normal color tends to a greyish-red, the centre of the papilla assuming a more whitish shade. Pulsation of the veins in the papilla in the dog is a singular condition revealed by opthalmoscopic examination.

The opthalmoscopic examination of the diseased eye showed that the head (bulb of the optic nerve) was so swollen that it could be easily seen in all its outlines without the aid of a corrective glass, while the picture of the retina and choroidea were quite indistinct. Its color was greyish white, but its peripheries were not distinctly marked from adjacent tissues.

The veins were the only vessel, of those running over the surface of the bulb, that could be distinctly made out. Their calibre seemed normal, but they appeared as if ascending the edges of the bulb almost perpendicularly, and were lost as abruptly in its tissues. The latter point is of diagnostic value, as it distinctly indicates the mush-room headed character of the bulb. In the same way, the veins of the bulb also show a paralletic displacement not so distinctly marked in the appositional portions of the retina. The veins suffered no change in their course in the middle and equatorial portions of the retina. The arteries are very filiform, and could only be followed in the perpendicular picture. They also ascend the edges of the bulb in a perpendicular manner; their course seems at times to be interrupted on the papilla, while in the retina it could be distinctly followed. Grouped around the centre of the bulb were to be seen four or five extremely delicate vessels forming a sort of wreath and then disappearing in its tissues. Pulsation of the vessels was not to be seen. The other portions of the retina and choroidea were normal. The changes in the right eye were of the same nature, though not so fully developed as in the left.

Another case is reported by the same author, in which the result of the examination agreed with those already quoted, except that they were more intense in character. "*A case of bilateral neuroretinitis in the dog*" is also reported by Dr. Sclanpp*.

HISTORY.

The patient is ten years old, and until recently has always appeared to be in perfect health, but within six to eight weeks the owner reports that the dog has emaciated very rapidly; a weak cough with emesis having also developed, which gave occasion to calling in professional advice.

Status presens: The patient is a powerfully built but very much emaciated individual; the visible mucosæ are very anæmic; the temperature and condition of the nose varying between warm and dry and cold and wet at times; the eyes withdrawn in the orbits. Thorax distended; abdomen very pendulant. Anasarca of the posterior limbs. Crural pulse not to be felt. Heart beat weak and fluttering. Temperature, morning, 39.1° C. Evening, 39.3°. The respirations were 24. 30 per minute, and of the abdominal type. The inspiration occupied three times as much time as the expiration. The cough seemed painful to the animal. An examination of the chest revealed nothing very abnormal, except bronchial rales. Appetite poor. Fœces, yellow brown in color, and enclosed some proglotids of T. cucumerina. Urine, dark yellow, clear, without sediment, Sp. grav. 10.22; reaction sound. Movements uncertain. An examination of the eyes revealed the following: Protecting parts intact. Conjunct. palp. et sclerae, anaemic: tension of the bulb normal. Pupil of either eye 4 mm. wide, reaction very slow, even to intensive light. Refracting media clear and without any recognizable changes.

---

* Ibid, Vol. II. Page 120.

Observation of the papilla gave occasion to three conclusions indicative of disease of the head of the optic nerve.

1st. Its form and size: in both eyes it varied essentially from its normal right-angled-triangular form. The angles, or points were decidedly obtuse, its form being more oval than anything else. It appeared to be almost double its natural size; its peripheral outlines were not sharply defined, but appeared gradually lost in surrounding tissues.

2d. The color of both papillae is an evenly distributed white with a slight tinge of greenish-yellow.

3d. The most striking phenomena, however, is the deportment of the blood vessels, but as they are almost identical with those already described it is unnecessary to detail them again.

The changes in the papillae were similar in each eye. The remaining parts of the retina and choroidea were apparently normal.

Ophthalmoscopic Appearance of the Papilla in the Dog, with the blood vessels in neuroretinitis.

The clinical diagnosis would then be: Heart disease, anasarca, dropsy of the different cavities of the body, bronchitis catarrhalis, and bilateral congestion of the papillæ.

### Autopsie.

Body much emaciated. Paniniculus adiposus almost entirely wanting. Visible mucose anaemic. Oedema of the posterior extremities from the tarsi down. On opening the abdomen we removed ten and a half quarts of water, the sp. grav. of which was 1031. The diaphragm was pushed out posteriorly. The situs of the abdominal contents normal. Stomach some-

Normal Papilla in the Eye of a Dog.

what tympanitic and contained but little digesta. The intestinal mucosa in catarrhal condition. A small amount of firm fœces in rectum.

The liver hypertrophied, of a bluish red color, the periacinous tissue hyperplastic and to be seen with naked eyes. When cut, the tissues of liver gave marked resistance to the knife. The gall-bladder soft, collapsed, and nearly empty.

Microscopic examination of the liver showed its cells to be compressed and lessened in number, the connective tissue in an active hyperplastic condition, and the capillaries, but still more the veins, to be changed into thick cord-like bodies filled with blood cells. Some appeared to have been burst, the contents being enclosed in cavities in the vicinity. The arteries were collapsed and empty.

Cirrhosis renalis on both sides. Bladder well filled. Prostate enlarged. The thorax contained but little fluid. The pericardial sack was distended by about a pint of fluid. The heart was very large and weighed 426 grns. Musculosis very pale, but intact. Valves shrunken and ostia stenotic. Coronary vessels normal. Bronchial mucosa swollen and covered with a watery-viscid secretion. Both lungs collapsed, of a pale yellow-red color ; resistant to the knife on cutting.

### Histological Examination by Dr. Westrum.*

The eyes were at once removed from the patient and carefully hardened in Miller's fluid for several months. On being cut equatorially the fundus revealed conditions corresponding exactly with the results of the intra vital opthalmoscopic examination.

The right-angled-triangular form of the papilla was most satisfactorily confirmed. The extension of the bulb into the posterior cavity was found to be correctly approximated, extending 1.1 mm. beyond the bend of the embracing retina.

The base of the papilla measured 3.5 mm.; and from the middle of its base in a direct line to the apex, 5 mms. The excavation in the body of the bulb approached much nearer the base of the triangle than the apex. Vessels upon the papilla were not to be seen, microscopically, but were visible in the retina. Perceptible changes were not to be made out either in the other part of the fundus or vitreous body.

### Microscopic Examination.

A power of 75 diameters showed an increase in the interstitial elements of the bulb as well as a curved projection of the net work of the lamina cribosa. A distinct widening of the space between the internal and external sheath of the nerve was well marked.

By a power of 610 diameters a distinct alveolar structure of the bulb was to be seen. A mass of cells was to be seen in the interstitial tissue surrounding these cavities, as well as numerous nerve-fibres of a very delicate structure. The bun-

---

* Ibid, Vol. 1, page 125.

dles of the lamina cribosa were strongly hypertrophied. The interstitial tissue of the optic nerve is also greatly augmented, the proliferation of its elements being essentially prominent in the central portions of the nerve. The vessels of the opticus also showed similar changes. Their walls were decidedly thickened, while in many places was found a contraction of their lumen, especially in vessels of medium size; a development of new capillaries had occurred on the most prominent parts of the bulbs.

The nerve fibres had an irregular, winding course, and were abnormally thin, and in many places had undergone molecular degeneration; they could be followed through the lamina cribosa into the head of the nerve for some distance. These fibres become less frequent here, and in the vicinity of the vitreous humor are no longer to be seen, that is on the convexity of the bulb. Crater-like indentations, corresponding to the furrows developed on the surface of the bulb by the opthalmoscopic examinations were to be seen.

The walls of these indentations had a marked lamellar construction, and one undoubtedly due to hyperplasia-sclerosis of the tissues of the neutral zone between the corpus vit., and the retina, the so-called membrana limitans interna, and the appositional elements of the corpus vit. A similar lamellar tissue in the same condition is also present in the remaining portions of the papilla which are in proximity to the vitreous body. The sheaths of the nerve showed important changes; both of them were eventually thickened and more dense—sclerosis. A decided proliferation of the cells of the endothelial lining of the external sheath, as well as upon the pial covering of the opticus was to be easily seen. Sometimes it appeared as a diffuse accumulation of nucleated cells of a roundish form, and again the cells were collected en mass; the picture presented much resembled that of the onion-like construction of cancroids. From their accumulation in circumscribed masses they frequently gave a nodulated appearance to the sheath. These cancroid formations varied much in size.

The changes in the retina varied much in size and form.

The swollen bulb loses itself gently in the surrounding retinal tissues, so that the elements of the latter have also the alveolar and lamellated structure already spoken of, in consequence of which the regular construction of the retinal elements is destroyed in the papillary zone. The layers and tissue immediately behind the nerve-fibre and ganglionic strata are completely destroyed ; the first being decidedly atrophied also.

The strata of nerve fibres in the retina is in all sections decidedly thinned and the ganglion cells but sparsely represented. A rather remarkable phenomenon is the exquisite prominence which the arcade-like, hypertrophied tissues, called the supporting fibres of Müller, show, made prominent by the atrophy of the nerve fibres and ganglion cells. Lacunae, or alveoloid cavities, are to be seen in all parts of this membrane, being mostly empty, but here and there crossed by rudiments of the fibres of the nervous tissues. As to the other portions of the retina, the picture varies much, some portions being undisturbed, while others show most serious disturbances, especially the median parts of the membrane. Deposits of molecular detritus are to be seen in various parts of the retina; they vary in color, from yellow to yellowish-brown—the last predominating ; they are especially prevalent in the nerve-ganglion layer around the vessels.

The choroidea shows many abnormal changes. The laminal-retina is often interrupted in its continuity, as well as the Tapetum cellulosa, which also suffer changes in thickness.

The choriocapillaries is not to be seen. Adhesions between the retina and choroid are frequent. The pigment cells of the latter have entirely perished in the diseased parts of the choroid ; the cells of the paranchyma in the immediate vicinity of these atrophied spots are notedly pigmented, hypertrophied, and of a very irregular form. Accumulations of pigment were frequent in the non-attached portions of the choroid ; the same could be easily mistaken for the pigmented cells of the paranchyma just mentioned. The choroid had become thinner than normal, its vessels thicker, their walls having a sclerotic appearance and lumen diminished. Other changes of importance were not noted.

## Figures and Tables

**Figure f1:**
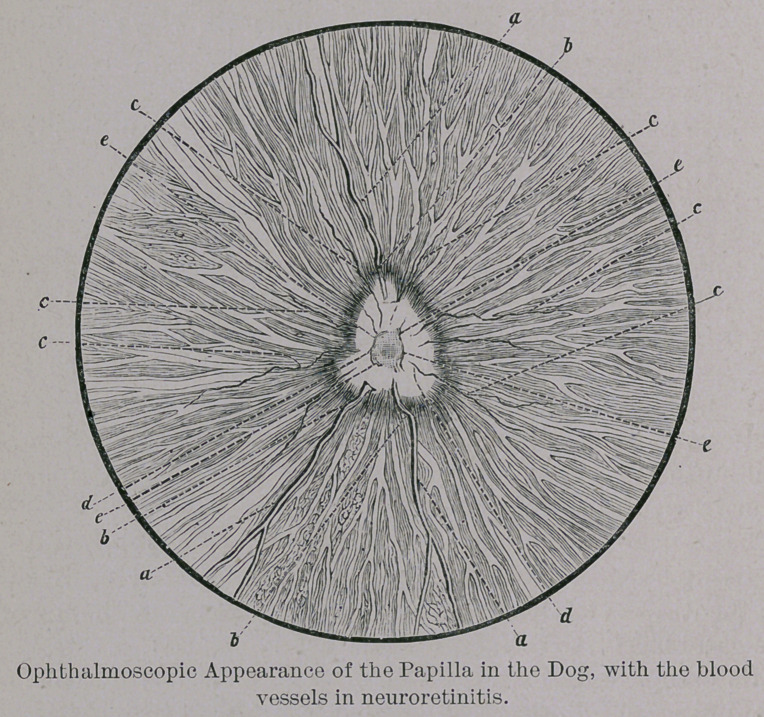


**Figure f2:**